# Critical Drip Size and Blue Flame Shedding of Dripping Ignition in Fire

**DOI:** 10.1038/s41598-018-34620-3

**Published:** 2018-11-08

**Authors:** Xinyan Huang

**Affiliations:** Research Center for Fire Engineering, The Hong Kong Polytechnic University, Kowloon, Hong Kong

## Abstract

Dripping of molten fuels is a widely observed fire phenomenon, and, by igniting other fuels, it can promote fire spread and increase fire hazards. In this work, dripping phenomena from fires of horizontally oriented wires, coated with polyethylene (PE), are investigated in the laboratory. It is found that as long as a flame is attached to the drip, thin tissue paper can be ignited by a single drip. Below a minimum diameter (*D*_*min*_ = 0.63 mm), the drip floats up. Above a critical diameter (*D*_*crt*_ = 2.3 mm), a flame can remain attached to the drip and ignite tissue paper as it falls through a distance of at least 2.6 m, thereby posing a significant fire hazard. A falling burning drip appears to the eye to be a blue chain of flame as a result of persistence of vision. Photographic evidence identifies a flame-shedding process, most likely associated with continual sequential ignition of fuel vapor within a von Karman vortex street generated behind the falling burning drip. The frequency of flame shedding agrees with both the frequency of modeled vortex shedding and the frequency of the unexpected sound that is heard during the process. This is the first time that combustion characteristics of dripping fire phenomena have been studied in detail, and this helps to better evaluate the risk and hazards of wire and façade fires.

## Introduction

**Figure 1:**
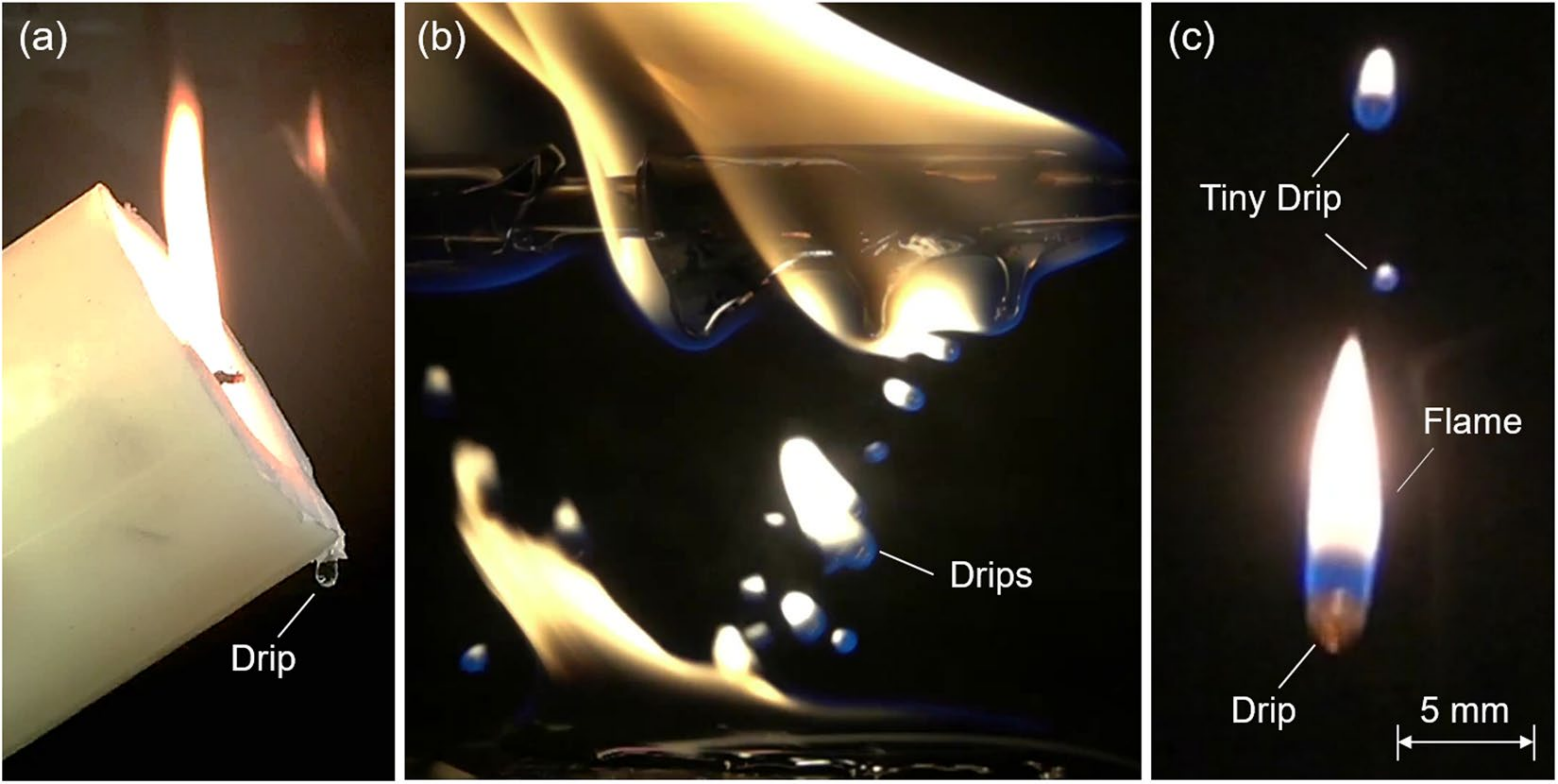
The dripping phenomenon in (**a**) the candle flame, (**b**) the electrical wire fire where the wire diameter is 8 mm and horizontal wind is 0.5 m/s, and (**c**) drips with flame (see Video 1 in the supplemental material).

The dripping of molten fuels is a widely observed fire phenomenon. The most common example of dripping can be found in the candle flame^1^, as shown in Fig. 1(a). Dripping also often occurs in the wire fire^2^ and façade fire^3^ where the thermoplastics, such as polyethylene (PE), polyvinyl chloride (PVC), polypropylene (PP), and expanded polystyrene (EPS), are widely used as the wire insulation, electrical devices, and insulation layer of façade panels. The dripping of melts is formed under the heat of the flame, and the flame is sustained by the pyrolysis gases from melts.

Under the gravity force, dripping takes place in the form of a continuously downward flow over fuel or discrete drips detached from fuel. Unlike the drip from the candle which is difficult to sustain a flame, the drip of thermoplastic can often carry a flame, posing a significant fire hazard. Figure 1(b,c) shows the dripping phenomenon in an electrical wire with PE insulation, where drips are continuously produced from the fire, and they can even sustain a pool fire of molten PE on the ground. It is apparent that dripping could play a vital role in the development and spread of fire, so there is an urgent need to understand the fire risk and combustion phenomena of dripping.

There are several experimental studies addressing the dripping phenomena during wire fires^2,4–6^ and façade fires^7,8^ as well as the standard tests like UL 94^9,10^ and ASTM D2863^11,12^. The size of drip and intensity of dripping depend on the polymer and the external heating^13–15^. Near the extinction limit, dripping removes the fuel and acts as the heat sink to promote the flame quenching^4^. The downward dripping flow acts as a heat source to increase the flame spread^2,5^. For the fire attached to a horizontal wire, the mass of drips detached was found to be 2~5 mg^16^. The frequency of dripping in wire fire increased as the AC frequency or overload current through the core was increased^6,17^. The intensity of dripping flow (i.e., the flooring) also has a significant impact to the development of façade fire^7,8^. Note that the dripping phenomena in fire only take place under gravity. In the microgravity environment like spacecraft, the melts would not flow away from the flame, but form a spherical shape under the surface tension force^18,19^. Only limited numerical works have simulated the dripping behaviors in the wire^20,21^ and façade panel^10^, but most of them did not include the condensed-phase pyrolysis and gas-phase flame, because of the complexity.

So far, we still have a very limited understanding of the dripping phenomena, particularly the ignition risk of dripping with flame and the combustion characteristics of polymer droplet. This work conducts a well-controlled experiment to provide more insights on the ignitability of a single PE drip with flame attachment, the effect of drip size on fire risk, and the special flame behaviors during the dripping process.

## Experiments

### Experiment design

For the most molten thermoplastics, both the viscosity and surface tension decrease significantly as the temperature is increased. Therefore, a temperature, which is much higher than the melting point, is required to achieve a good mobility of melts and allow the melts to form a drip and then get detached. One criterion for dripping to occur is that the gravity of the accumulated molten ball must exceed its surface tension force. Therefore, the mass of a drip (*M*_dr_) or the Bond number (*Bo*) should satisfy

1a $${M}_{dr}g={\rho }_{dr}(\frac{\pi }{6}{D}^{3})g\ge {\sigma }_{dr}(\pi D)\,{\rm{or}}\,Bo=\frac{{\rho }_{dr}\,g{D}^{2}}{{\sigma }_{dr}}\ge 6$$

where *g* = 9.81 m/s^2^ is the gravity acceleration, *ρ*_*dr*_, *D*, and *σ*_*dr*_ are the bulk density, diameter, and surface tension of the drip or molten ball, respectively.

For PE, the required temperature could be above 500 °C which is higher than the temperature of pyrolysis or piloted ignition (*T*_*py*_ ≈ 400 °C). In other words, when PE is hot enough to drip, it will start to pyrolyze and release a large amount of smoke. Once produced, the small drip will quickly lose its mass via strong pyrolysis if overheated, or quickly cool down by the environment and lose its mobility if underheated. Moreover, there is a large uncertainty to measure the temperature of a small drip (diameter below 3 mm) and a non-uniform temperature via the thermocouple or the infrared camera. In fact, without igniting the PE, it is extremely difficult in the experiment to produce a molten drip and make it hot enough to flow and detach.

**Figure 2:**
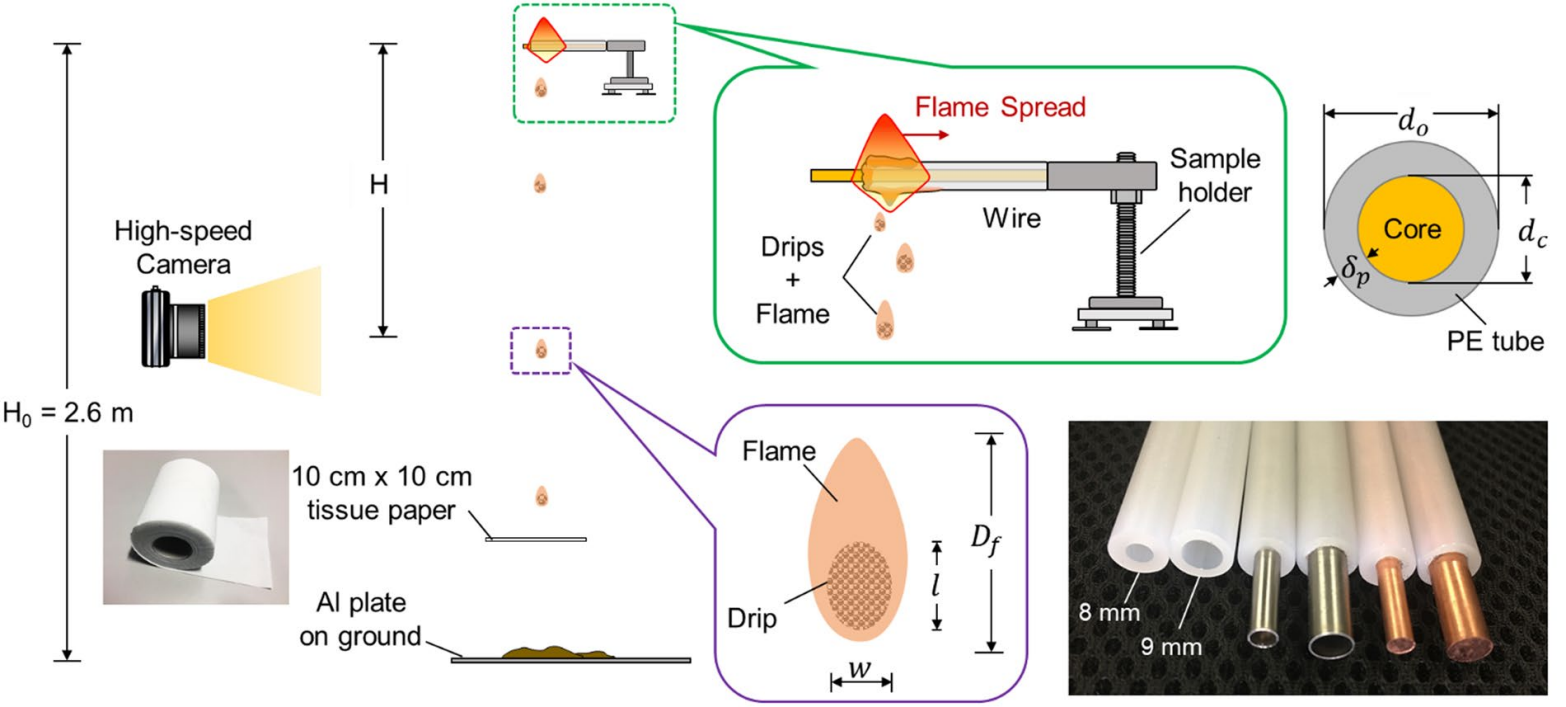
Experimental setup for the dripping ignition of tissue paper, where drips are produced from a burning wire.

In this work, drips are produced from a burning PE tube that is placed horizontally, as illustrated in Fig. 2. The experimental setup is the same as the past work of studying the rate of flame spread in PE wires^2,5^. To better control the experiment, the solid copper (Cu) rod or thin-wall stainless steel (SS) tube is inserted into the PE tube to modify the temperature of molten PE within the flame. Then, the size of dip can be controlled, as suggested by Eq. 1(a). Two tested PE tubes have the outer/inner diameter of 8.0/3.5 mm and 9.0/5.5 mm, respectively (see detailed configurations in Fig. 2 and Table 1). The length of the PE tube is 10 cm. The burning PE is placed 30 cm below the ceiling and 2.6 m above the floor, allowing drip to fall up to 2.6 m.

**Table 1:** Characteristics of drip for different wire configurations.

Case	1	2	3	4	5	6
core material	No core		SS		Cu	
core diameter, *d*_c_ (mm)	3.5	5.5	3.5	5.5	3.5	5.5
outer diameter, *d*_o_ (mm)	8.0	9.0	8.0	9.0	8.0	9.0
thickness of PE tube, *δ*_*p*_ (mm)	2.25	1.75	2.25	1.75	2.25	1.75
mass of drip, ${\overline{M}}_{dr}$ (mg)	2.5 ± 0.2	2.6 ± 0.2	3.0 ± 0.2	4.1 ± 0.5	4.6 ± 0.4	5.0 ± 0.4
diameter of drip, $\overline{D}$ (mm)	1.9 ± 0.2	1.9 ± 0.2	2.1 ± 0.2	2.3 ± 0.2	2.5 ± 0.3	2.6 ± 0.3
bulk density, *ρ*_*dr*_ (kg/m^3^)	700	702	620	640	560	540
porosity, *ψ* = *ρ*_*dr*_/*ρ*_*PE*_ (−)	0.73	0.75	0.64	0.67	0.59	0.57
extinction height, ${\overline{H}}_{ex}$ (cm)	20	22	33	38	40	42
probability of flame attachment, *P*_*f*_	0%	0%	20%	39%	56%	67%
terminal velocity, *V*_*T,cal*_ (m/s)	3.6	3.6	3.8	3.9	4.1	4.2

To quantify the ignitability of a drip, a double-layer cellulose tissue paper of 10 cm × 10 cm is placed at different heights to capture only one drip and see whether the ignition occurs. This thin tissue paper is chosen because it is a common residential fuel and is very easy to ignite. The ignition is defined as that a flame can be sustained in the paper and eventually burn out the paper. Smoldering ignition by a single drip is not observed in the experiment. To determine the ignitability of a single drip, at least 10 repeating tests are conducted.

### Characteristics of drip

To observe the drip and the dripping process, a high-speed camera (Sony DSC-RX10M3) up to 960 fps is placed at different heights. Because the drip is produced from the PE flame, initially there is a flame attach to the drip. To observe the dripping flame better, experiments are conducted in the dark room. To help locate the drip in each frame, a linear LED light is placed vertically parallel to the dripping projection as the backlight. The size and shape of drip are measured when the drip is just detached from the wire with a small falling velocity. To determine the location and velocity evolution of each drip, videos are processed frame by frame using an in-house MATLAB program. Because the drip is not a perfect sphere but more like an ellipsoid (see Figs 1 and 2), its length (*l*) and width (*w*) are measured, and its characteristic diameter is defined as *D* = (*w*^2^*l*)^1/3^ throughout the paper.

The drips produced from the burning PE tube cannot be identical due to the complexity of fire experiment. Thus, more than 100 drips, which are produced from at least five wires of the same configuration, are measured to determine each parameter. To measure the mass of drip, an aluminium Petri dish is placed 20 cm below the wire to capture 10 continuous drips and quickly quench the flame. Then, the mass gain of Petri dish is measured by an analytical balance with a precision of 0.01 mg. Table 1 summarizes the characteristics of different drips where the overall uncertainty is about 10%.

**Figure 3:**
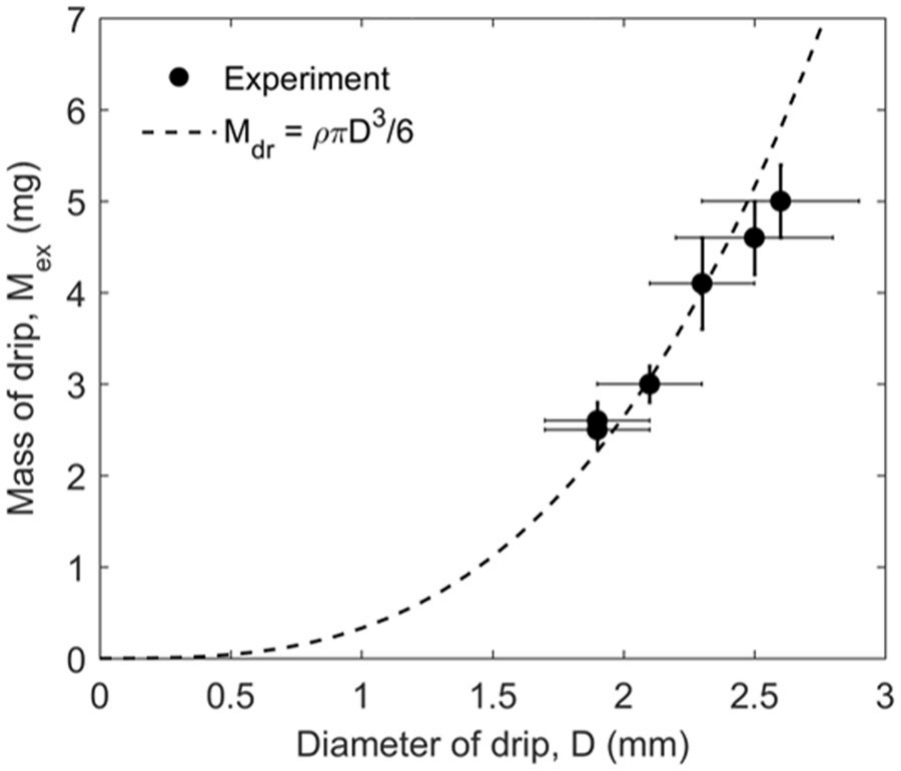
Mass (${\overline{M}}_{dr}$) and diameter ($\overline{D}$) of drip where ${\overline{\rho }}_{dr}=630$ kg/m^3^ is used in the calculation.

Figure 3 further shows the measured mass (${\overline{M}}_{dr}$) and diameter ($\overline{D}$) of drip produced from the burning PE. It can be seen that the mass and size of drip increase with the thermal conductivity of the core material, because the core can cool the melts and increases its surface tension (*σ*_*dr*_) in Eq. (1a). Also, the measured drip mass is relatively uniform, and its standard deviation is no more than 10%. Note that the calculated density of drip (*ρ*_dr_) is smaller than the literature value of molten PE (*ρ*_*PE*_ = 960 kg/m^3^). In fact, the drip is porous, as there is a clear bubbling process inside the drip and it is continuously heated by the surrounding flame. The porosity of drip (*ψ* = *ρ*_*dr*_/*ρ*_*PE*_) is estimated and listed in Table 1 as well.

## Results

### Ignitability of drip

**Figure 4:**
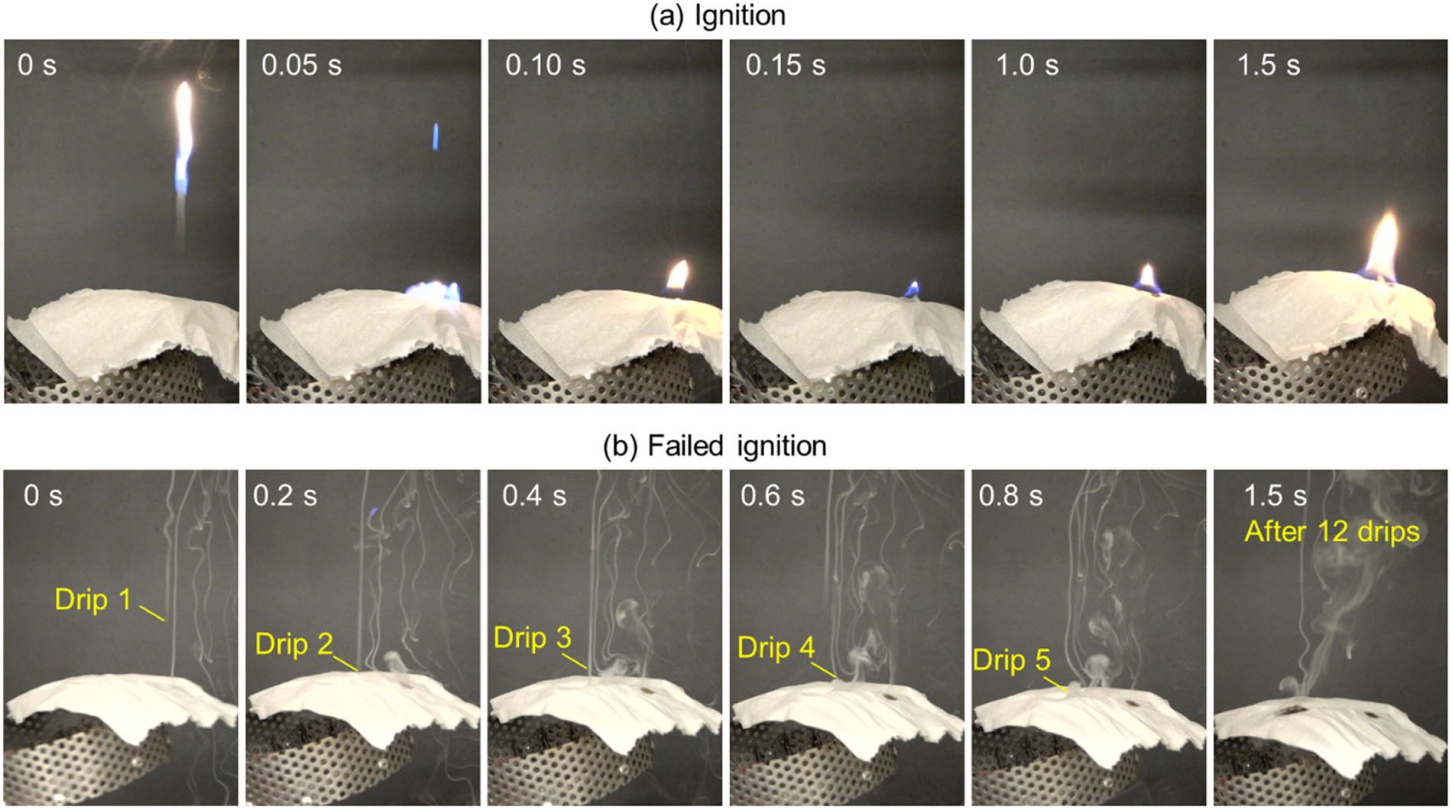
Snapshots of (**a**) a successful ignition of tissue paper by a single drip (${\overline{M}}_{dr}=5.0\,{\rm{mg}}$ mg, $\overline{D}$ = 2.6 mm) with flame, and (**b**) a failed ignition of tissue paper by multiple drips without flame (${\overline{M}}_{dr}$ = 2.5 mg, $\overline{D}$ = 1.9 mm). More details can be viewed from Videos 2 and 3 in the Supplemental Material.

Figure 4 shows a typical process of (a) a successful ignition of tissue paper by a single drip with flame attachment, and (b) a failed ignition of tissue paper by multiple drips without flame (also see Videos 2 and 3 in the Supplemental Material). It is found that regardless the height between the wire and paper (*H*), as long as the flame is attached to the drip when it reaches the paper, the thin tissue paper can be ignited by a single drip. On the other hand, if the flame is not attached to the drip, no flaming ignition is found in tissue paper even when it is continuously hit by multiple drips.

There are two major reasons, (1) the pyrolysis temperature of cellulose paper is blow 300 °C^22^ which is much lower than the pyrolysis temperature of PE (~400 °C), so the drip is hot enough to initiate the pyrolysis of paper, and (2) the tissue paper can absorb the molten PE like that the candle wick can absorb the molten wax, so the flame attached to the drip can be sustained on paper. On the other hand, without a flame attaching to the drip, even if the drip can make the paper pyrolyze, ignition is not possible without a pilot source. Therefore, *the probability of paper ignition at a specific height is equal to the probability of flame attachment to the drip at the same height*.

**Figure 5:**
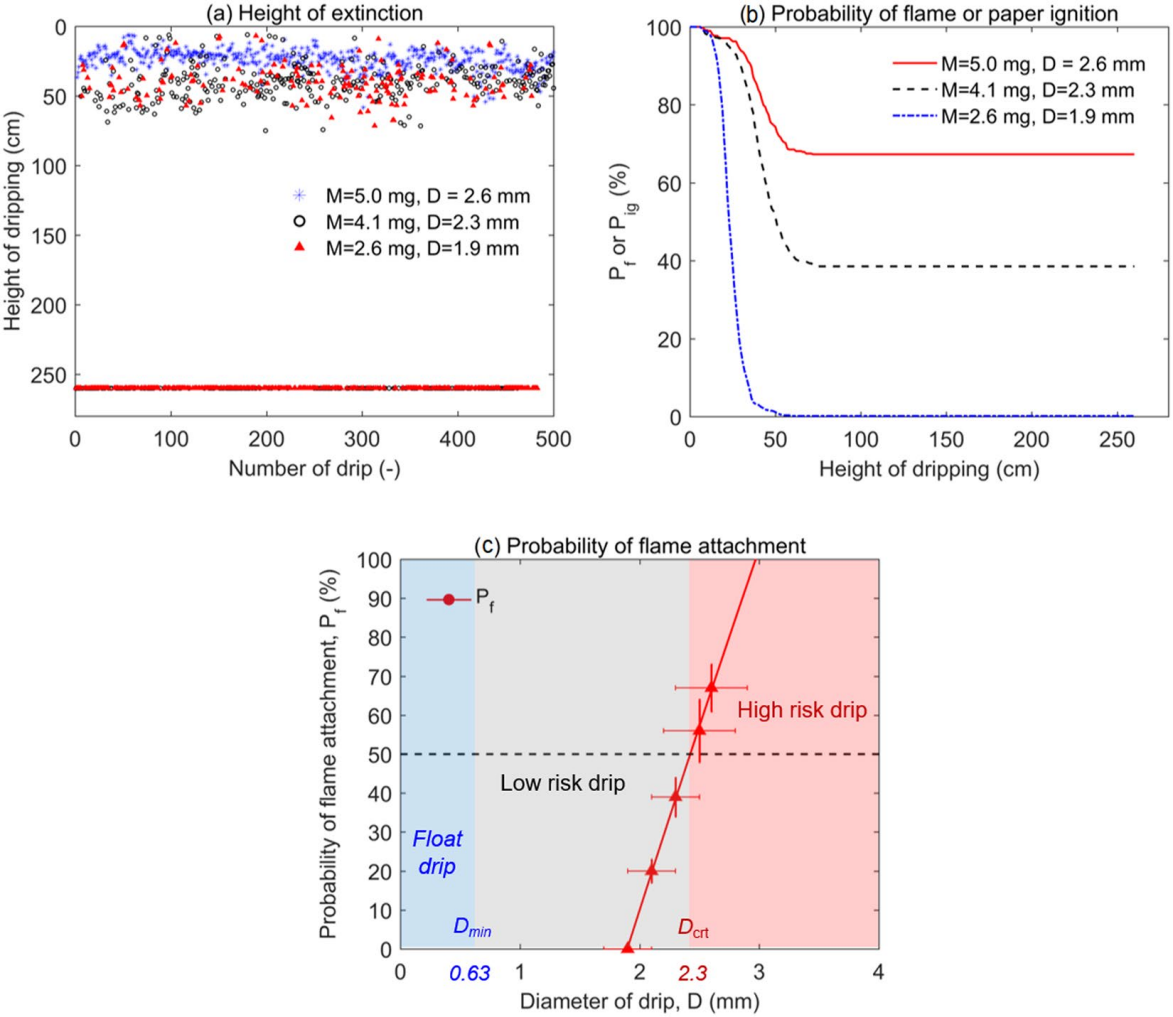
(**a**) The height of extinction (*H*_ex_) for different drip sizes, (**b**) the probability of paper ignition (*P*_*ig*_) by a single drip as a function of dripping height, and (**c**) the probability of continuously flame attachment (*P*_*f*_) for a 2.6-m fall varying with ${\overline{M}}_{dr}$.

Note that there are tiny drips that do not fall but float up (discussed more in later section). These tiny drips are not included in calculating the ignition probability, because they quickly burn out and never reach the paper. Figure 5(a) shows the height when the flame of drip extinguished for three different sizes of drip, where 500 drips are measured for each size. Because the size of drip cannot be perfectly controlled, a large data scattering is expected. Nevertheless, results still show that there is a critical height for flame extinction (*H*_ex_) which depends on the size of drip. The mean value of this critical height (${\overline{H}}_{ex}$) for each drip size is listed in Table 1, and it increases from 20 cm to 42 cm as the mass (or size) is increased from 2.5 mg to 5.0 mg (or from 1.9 mm to 2.6 mm). More importantly, if the flame is not extinguished within *H*_ex_, it will continue to follow the drip until the drip reaches the ground (2.6 m). The dripping process can be viewed in Videos 4–8 in the Supplemental Material.

Figure 5(b) replots all the data of three drip sizes in Fig. 5(a) and shows the probability of flame attachment (or the ignition probability of tissue paper) as a function of dripping height. For the smallest drip (${\overline{M}}_{dr}$ = 2.6 mg), surprisingly none of the drips can carry the flame to reach the ground (see Video 7). For larger drips (${\overline{M}}_{dr}$ = 4.1 mg) and (${\overline{M}}_{dr}$ = 5.0 mg), 39% and 67% of drips can carry the flame to reach the ground and ignite the tissue paper (see Video 4). Figure 5(c) plots the probability of continuous flame attachment (*P*_f_) for a fall of 2.6 m as a function of drip mass (${\overline{M}}_{dr}$). By defining the 50% probability as the characteristic value, we can find the critical drip mass of 4.4 mg and the critical drip diameter of 2.3 mm, which separates low-fire-hazard and high-fire-hazard PE drips. In other words, for a drip from the wire fire or façade fire where PE are often used as insulation material, if it is larger than this critical size, it can carry the flame to ignite the fuel at least in the floor below, posing a significant fire hazard of vertical fire spread. Due to the limitation of floor height, a drip height larger than 2.6 m (for multiple floors) cannot be tested in this work.

### Flame shedding behind the drip

**Figure 6:**
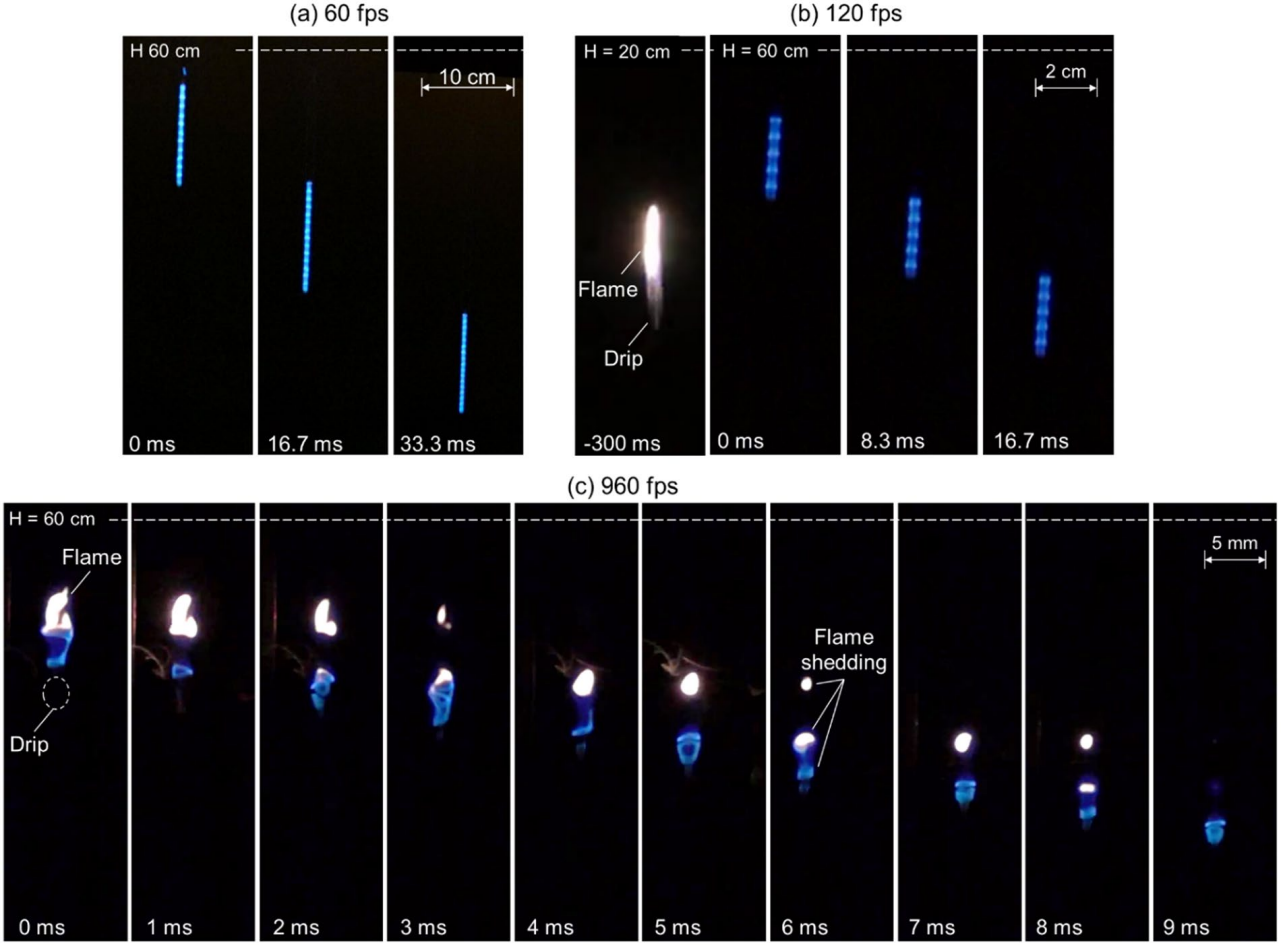
Snapshots of drip (${\overline{M}}_{dr}$ = 5.0 mg, $\overline{D}$ = 2.6 mm) with flame under shutter speeds of (**a**) 60 fps, (**b**) 120 fps, and (**c**) 960 fps. See Videos 4–6 in the supplemental material.

Figure 6 shows the snapshots of the flame attached to the relatively large drip (${\overline{M}}_{dr}$ = 5.0 mg, $\overline{D}$ = 2.6 mm) under different shutter speeds. Interestingly, the dripping flame looks completely differently under different shutter speeds, from the “*blue chain flame*” at the low shutter speed to “*flame shedding*” at the high shutter speed. In the eyes of the experimenter, a “blue chain flame” is also observed for the drip during the falling of 2.6 m. Because of the persistence of vision, the observed “blue chain flame” by the human eyes and the camera is essentially an illusion. In other words, the flame of drip varies in a frequency much faster than the response time of human eyes and the shutter speed of a regular camera.

Under the high shutter speed of 960 fps, the detailed evolution of flame structure can be revealed, as shown in Fig. 6(c). It seems that the blue flame continuously peels off from the drip, just like the classical von Karman vortices, and then becomes a bright yellow flame. Such shedding process of flame is very stable, and it will fall with the drip to the ground 2.6 m below for about 1 s and is accompanied with unexpected sharp sound (discussed more in later section). During the whole dripping process, there is no smoke, if the flame is attached to the drip. To the author’s best knowledge, such flame shedding has not been observed before. Previously, the flame of a porous spherical gas burner with the diameter of 6 mm had been investigated, and the minimum flow velocity (*Re*_min_ = 138) for a stable wake flame was determined^23^. Later, it was found that for a porous spherical of 12.2 mm diameter, the wake flame was still stable under an uprising airflow of 6 m/s (*Re* = 13,200)^24^. Neither of these works had observed a similar phenomenon of “*flame shedding*”.

**Figure 7:**
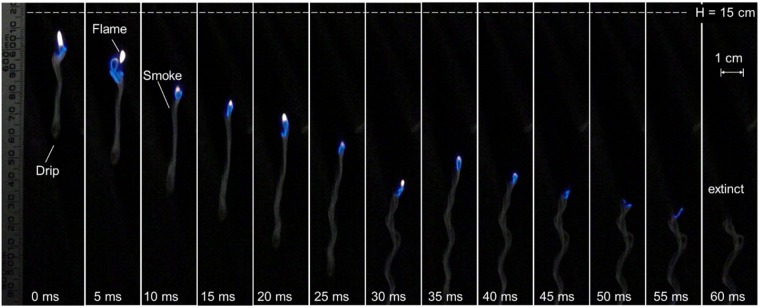
Snapshots of drip (${\overline{M}}_{dr}$ = 2.5 mg, $\overline{D}$ = 1.9 mm) without flame under shutter speeds of 960 fps. See Videos 8-9 in the supplemental material.

Figure 7 shows a typical extinction process of drip flame when the drip is relatively small (${\overline{M}}_{dr}$ = 2.5 mg, $\overline{D}$ = 1.9 mm). The flame is similar to a lifted diffusion flame that is burning the pyrolysis gas (smoke) released from the drip. Compared to the case with flame attachment in Fig. 6(c), the gap between drip and flame is much larger, and it continuously increases. From 30 ms to 40 ms, the flame even contemporarily moves up. In other words, the flame is not able to catch up with the drip or heat up the drip. Eventually, the flame extinguishes as air significantly dilutes the pyrolysis gas. In the case of extinction, a large amount of smoke can be observed, and the smoke is essentially the pyrolysis gas from the drip. In other words, the drip is still hotter than its pyrolysis point, which agrees with the bubbling phenomenon found inside the drip.

### Velocity profile of drip

**Figure 8:**
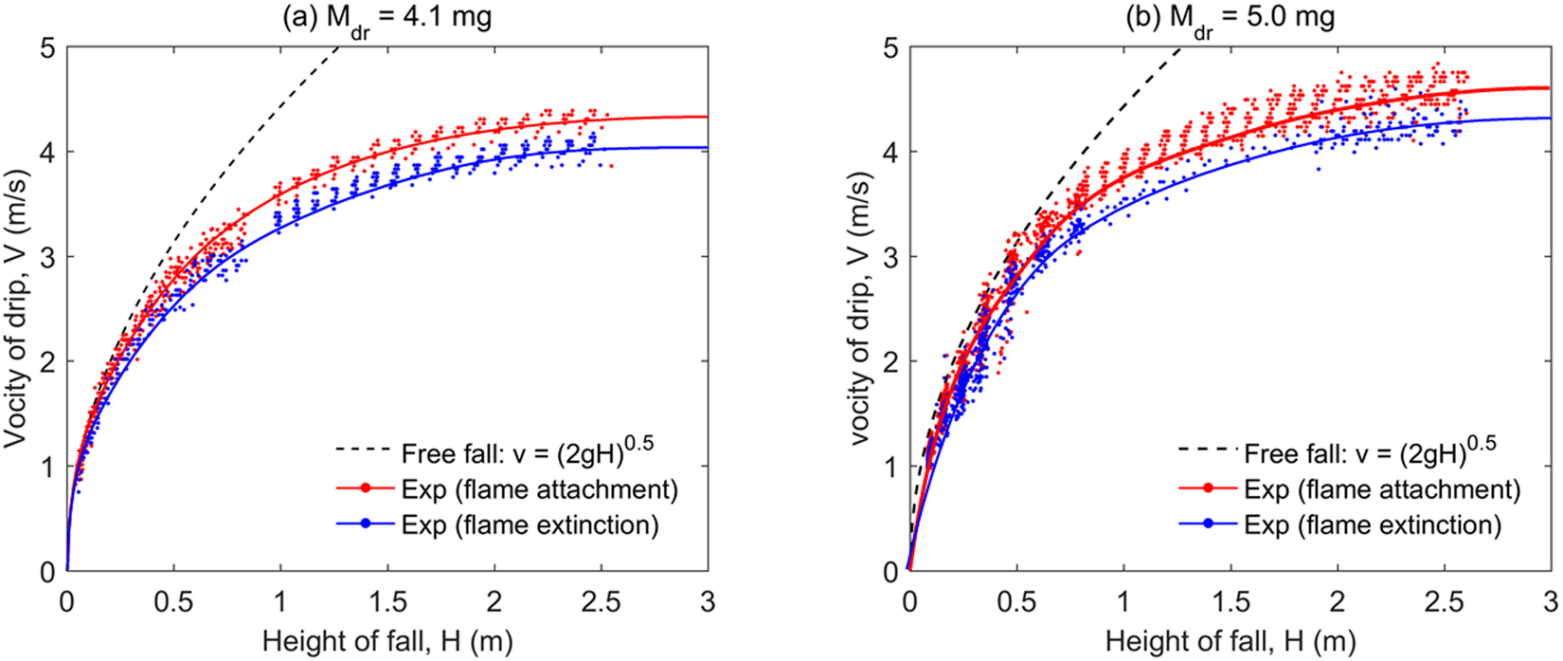
The measured falling velocity of drip varying with height for the mass of drip (**a**) ${\overline{M}}_{dr}$ = 4.1 mg, $\overline{D}$ = 2.3 mm, and (**b**) ${\overline{M}}_{dr}$ = 5.0 mg, $\overline{D}$ = 2.6 mm.

The entire dripping process is very short. For a drip of ${\overline{M}}_{dr}$ = 4.1 mg and $\overline{D}$ = 2.3 mm, it takes 0.95 ± 0.05 s to reach the ground 2.6 m below. By measuring the position of drip in each frame under a fixed shutter speed, the dripping velocity (*V*) at different heights (*H*) can be determined. Figure 8 shows the measured dripping velocity as a function of dripping height for drips with continuous flame attachment and drips with extinction. Because of the drag, the velocity profile of drip will deviate from the free-fall profile and eventually reaches the terminal velocity (*V*_*T*_). As limited by the maximum dripping height of 2.6 m, the terminal velocity is not completely achieved, but the trend of reaching a terminal velocity is evidential in Fig. 8.

The terminal velocity can be estimated from the balance between drag and gravity as

1b $${M}_{dr}g={\rho }_{dr}(\frac{\pi }{6}{D}^{3})g=\frac{1}{2}{C}_{D}{\rho }_{g}{V}_{T}^{2}(\frac{1}{4}\pi {D}^{2})$$

where *ρ*_g_ are the gas density, and the mass loss due to pyrolysis is neglected. The drag coefficient (*C*_D_) can be estimated by the Oseen’s approximation

2 $${C}_{D}=\frac{24}{{\rm{Re}}}(1+\frac{3}{16}{\rm{Re}})$$

3 $$\mathrm{Re}=\frac{{\rho }_{a}{V}_{T}D}{\mu }$$

where *μ* is the dynamic viscosity of pyrolysis gas because the drip is surrounded by the pyrolysis gas. Then, the terminal velocity (*V*_*T*_) is calculated using *ρ*_*dr*_ = 640 kg/m^3^, *ρ*_*g*_ = 0.27 kg/m^3^ (air at 1300 K), and *μ* = *μ*_*F*_ = 3.27 × 10^−5^ kg/m-s, as listed in Table 1. As the diameter of drip increases from 1.9 mm to 2.6 mm, the calculated terminal velocity increases from 3.6 m/s to 4.2 m/s, which agrees well with the trend of experimental data in Fig. 8. The calculation slightly under-estimates the terminal velocity mainly because the shape of drip is not a perfect sphere but an ellipsoid.

Figure 8 further shows that for a drip with continuous flame attachment, both its dripping velocity under the same dripping height and the projected the terminal velocity are slightly larger than those for a drip with extinction, despite of the data scattering and overlapping. It further supports the conclusion that there could be a critical drip size, above which the drip can carry a flame to ignite the fuel in floors below, i.e. a significantly larger fire hazard.

## Discussions

In the experiment, many tiny drips are produced when the tail of the main drip breaks down, and they are also accompanied by flame (see Fig. 1 and Videos 1 in the supplemental material). When the drip is very small and surrounded by a flame, it is found to either directly float up or fall and burn for a distance and then float up, thus, defining a minimum size for dripping (*D*_*min*_). Therefore, in total three regions can be defined based on the size of drip, as seen in Fig. 5(c):

1. *D* < *D*_min_ (floating drip): Tiny drip will float up and quickly burn out, i.e., a *negligible* fire risk;
2. *D*_*min*_ < *D* < *D*_crt_ (low-risk drip): dripping flame cannot fall for more than 0.7 m, i.e., a *low* fire risk;
3. *D* > *D*_crt_ (high-risk drip): dripping flame can fall for more than 2.6 m, i.e., a *high* fire risk.

### The minimum size for dripping (D_min_)

To allow a drip inside a flame to fall down (i.e., the downward velocity should be negative, $\overrightarrow{V} < 0$) rather than floating up ($\overrightarrow{V} > 0$), the value of its terminal velocity (*V*_*T*_) must be larger than that of the buoyancy flow velocity (*V*_*b*_) introduced by the flame. In other words, the absolute velocity (*V*) of dripping^25^ should satisfy that

4a $$V={V}_{b}-{V}_{T}\le 0$$

For such a tiny drip, its Reynolds number is small (*Re* < 1). Then, the Oseen approximation can be use^26^, and the drag coefficient (*C*_*D*_) becomes

4b $${C}_{D}=\frac{24}{{\rm{Re}}}(1+\frac{3}{16}{\rm{Re}})\approx \frac{24}{{\rm{Re}}}$$

where the propulsion force due to drip evaporation is neglected. Then, the terminal velocity increases rapidly with the square of drip diameter (*D*) as

4c $${V}_{T}=\frac{{D}^{2}}{18}\frac{{\rho }_{dr}}{\mu }$$

where *μ* is the viscosity of gas inside the flame attached to the drip.

The upward buoyancy flow velocity introduced by the flame of drip may be estimated as

5 $${V}_{b}\approx \sqrt{2g{D}_{f}}$$

which increases slowly with the square root of flame diameter (*D*_*f*_). The flame diameter may be estimated by the mass transfer number (*B*)^27^ as

6 $${D}_{f}\approx D\frac{\mathrm{ln}(1+B)}{\mathrm{ln}\,[(1+\phi )/\phi ]}$$

7 $$B\approx \frac{{\rm{\Delta }}{H}_{c}/\phi +{c}_{g}({T}_{\infty }-{T}_{py})}{{\rm{\Delta }}{H}_{py}}$$

The burning of the plastic drip (or droplet) should also follow the classical *D*^2^ law^27^, so the lifetime of drip is

8 $${t}_{D}\approx \frac{{D}_{0}^{2}}{K}$$

The burning rate constant (*K*) is

9 $$K=\frac{8{\lambda }_{g}}{{\rho }_{dr}{c}_{g}}\,\mathrm{ln}(1+B)$$

For the ethylene flame, *ϕ* = 14.7 is the air-fuel stoichiometric ratio; Δ*H*_*c*_ ≈ 50 MJ/kg is the heat of combustion; *T*_∞_ = 300 K is the ambient temperature; *T*_*py*_ ≈ 700 K is the pyrolysis temperature of PE^28^; *T*_*f*_≈1900 K is the flame temperature; the average temperature between the flame and drip surface is *T*_*fp*_ = (*T*_*f*_ + *T*_*py*_)/2 = 1300 K; *c*_*g*_ = *c*_*F*_(*T*_*fp*_) ≈ 3.8 kJ/kg-K^29^; *λ*_*g*_ = 0.4*λ*_*F*_(*T*_*fp*_) + 0.6*λ*_*O*_(*T*_*fp*_) ≈ 0.1 W/m-K is the gas thermal conductivity^30^; the lowest density of the porous drip in Table 1 is *ρ*_*dr*_ ≈ 540 kg/m^3^; and Δ*H*_*py*_ = 1.8 MJ/kg is the pyrolysis (gasification) heat of PE. Therefore, we can estimate

10 $$\{\begin{array}{rcl}B & = & 1.05\\ \frac{{D}_{f}}{D} & = & 11\\ K & = & 2.85\times {10}^{-7}{{\rm{m}}}^{2}/{\rm{s}}\end{array}$$

Then, using the criterion for dripping, ${V}_{T}\ge {V}_{b}$,

11 $$\frac{{D}^{2}}{18}\frac{{\rho }_{dr}}{\mu }\ge \sqrt{22gD}$$

the minimum size for dripping at *V* = *V*_*T*_ − *V*_*b*_ = 0 is

12 $${D}_{{\rm{\min }}}={[22g{(\frac{18{\mu }_{f}}{{\rho }_{dr}})}^{2}]}^{\frac{1}{3}}\approx 0.63\,{\rm{mm}}$$

where *μ* = *μ*_*f*_(*T*_*fp*_) = 3.27 × 10^−5^ kg/m-s. Drip will float up if it is smaller than this size. At the same time, we can get the minimum terminal velocity (${V}_{T,{\rm{\min }}}$), minimum flame size for dripping (${D}_{f,{\rm{\min }}}$), and the maximum lifetime of floating (${t}_{D,{\rm{\max }}}$) as

13 $${V}_{T,{\rm{\min }}}=\frac{{D}_{{\rm{\min }}}^{2}}{18}\frac{{\rho }_{dr}}{{\mu }_{a}}=0.37{\rm{m}}{\rm{/}}{\rm{s}},\,{D}_{f,{\rm{\min }}}=7\,{\rm{mm}},\,{t}_{D,{\rm{\min }}}=\frac{{D}_{{\rm{\min }}}^{2}}{K}=1.4\,{\rm{s}}$$

**Figure 9:**
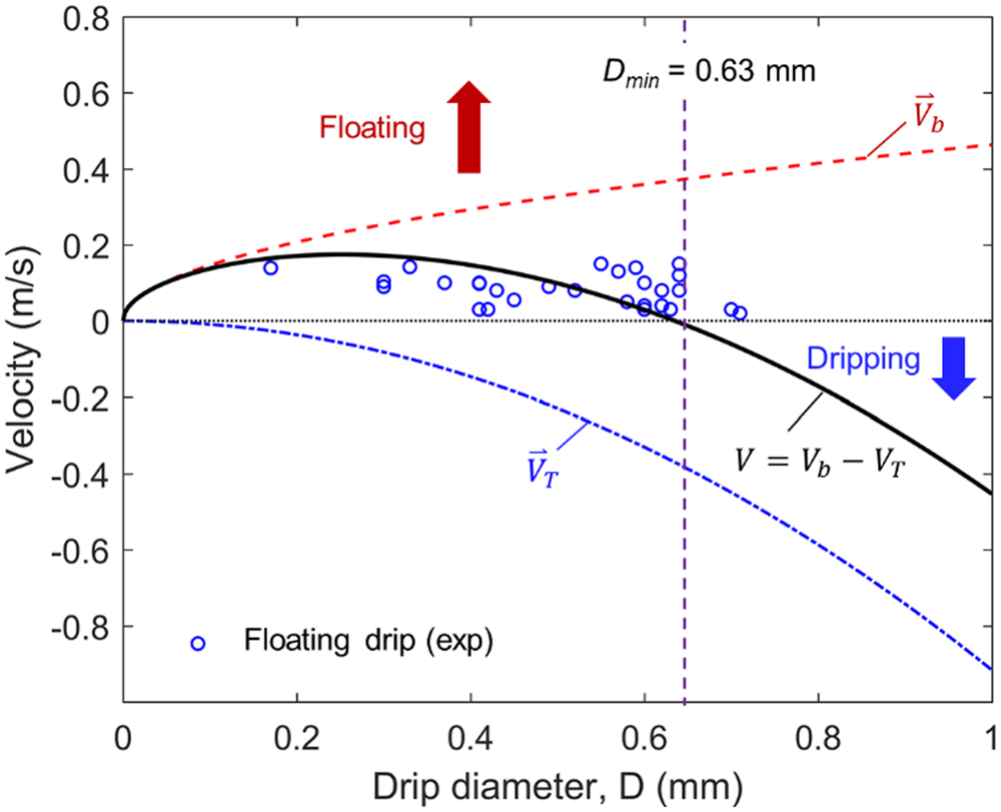
The calculated terminal velocity (*V*_*T*_), the upward buoyancy flow velocity (*V*_*b*_) and the minimum diameter for dripping (*D*_*min*_) where the final velocity of tiny drip before flame disappear is shown.

Figure 9 shows the calculated velocities (*V*, *V*_*T*_, and *V*_*b*_) as a function of drip diameter (*D*), where *V* < 0 for dripping and *V* > 0 for floating. The diameters of floating tiny drips are also measured and plotted against their final velocity right before the flame is extinguished due to burn out or blow off. Most of the floating drips in the experiment are found to be smaller than *D*_*min*_ = 0.63 mm, agreeing well with the theoretical calculation.

The floating of some slightly larger drips is also observed, when they are close to the flame on PE tube that can provide a much stronger uprising buoyancy flow. The measured lifetime of floating drip in the experiment is usually less than 1 s, suggesting that the drip may not completely burn out. It is probably because (1) the PE drip is more difficult to gasify with a high pyrolysis point (400  °C), compared to the boiling point of a liquid hydrocarbon (e.g. 98  °C for n-heptane), and (2) the buoyancy flow induced by the flame on PE tube is strong enough to blow off the tiny flame.

Note that the provided calculation is qualitative in nature because many approximations are used. For example, the flame of drip is not perfectly spherical under gravity, as shown in Fig. 1(c). Also, the composition of PE pyrolysis gases is more complex than pure ethylene. Pyrolysis experiment with fluidized bed reactor showed that the pyrolysis gas includes 37% ethylene, 24% methane, 19% propylene, 7% butylene, and other minor components^31^. Nevertheless, the physics behind the floating of tiny drips are well explained.

### Mechanism of flame shedding

**Figure 10:**
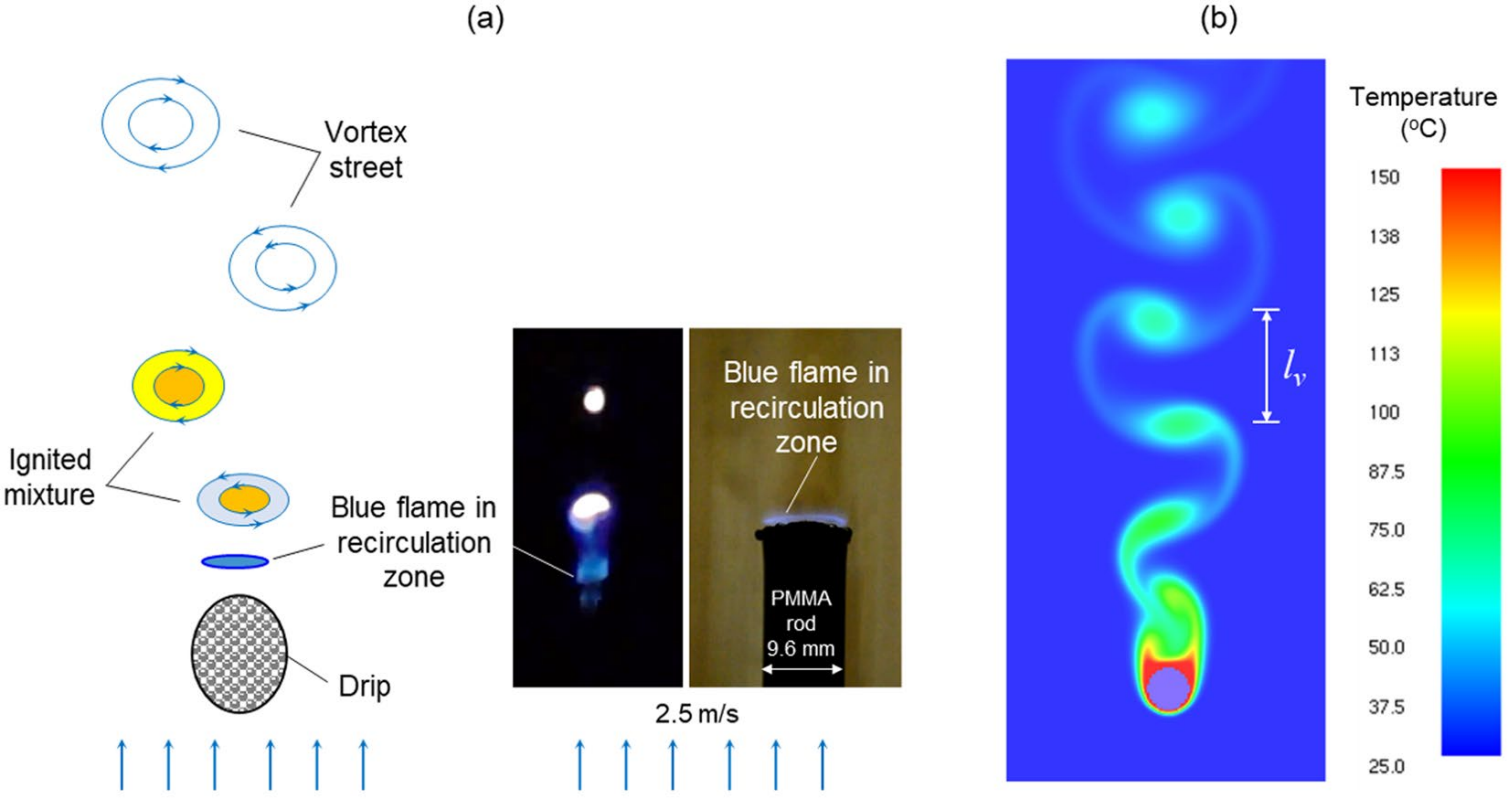
(**a**) Illustration of the vortex shedding behind drip and the similar the blue flame sheet attached to the recirculation zone above the top of PMMA rod^32^ (see Video 9 in the supplemental material), and (2) simulated von Karman vortex street behind a 2-D cylinder of 2-mm diameter.

One hypothesis is proposed for the observed “blue chain flame” under the low shutter speed or the “flame shedding” under the high shutter speed in Fig. 6. That is, *the flame shedding is the continuous ignition of von Karman vortex street generated behind the fast-falling drip*, as illustrated in Fig. 10(a).

As the dripping velocity increases with the dripping height, the vortex shedding will be generated above a critical velocity or *Re* Number. At the same time, the diffusion flame can no longer enveloping the entire drip, but move back to the recirculation zone right behind the drip, and eventually, the flame gets stabilized and becomes blue. Recently, such blue flame within the recirculation zone is also observed for burning a PMMA cylinder under a large opposed flow (>2.5 m/s)^32^ (see Fig. 10(a) and Video 9 in the Supplemental Material). The flame cannot be sustained outside the wake zone because of the large strain rate, and the fuel of pyrolysis gases cannot be completely consumed. Instead, the remaining fuel and air are mixed in the vortex. Once reaching the flammability limit, the vortex can be ignited. As vortices are continuously produced behind the drip, the ignition process is also continuous at the same frequency of vortex shedding. Because the frequency of vortex shedding increases with the velocity of drip, the ignition frequency also increases during the falling process.

**Figure 11:**
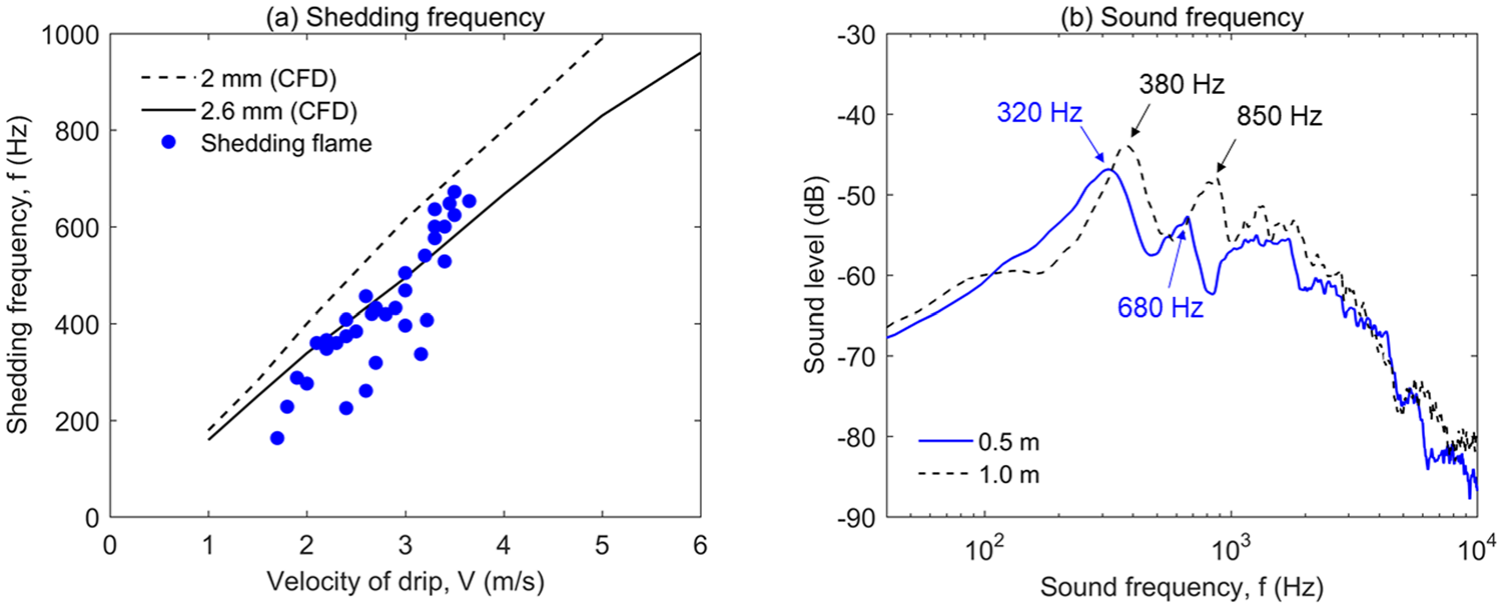
(**a**) The measured frequency of flame shedding and the simulated frequency of vortex shedding, and (**b**) the sound frequency spectrum during dripping (${\overline{M}}_{dr}$ = 4.1 mg).

Through the frame-by-frame video process, the frequency of flame shedding can be measured at different heights and velocities of drip, as shown in Fig. 11(a). As expected, there is a large scattering in the measured data, because of the limited view and shutter speed of the camera, the acceleration of drip, and the size difference of each drip. Despite the data scattering, the shedding frequency is found to increase with the velocity of drip.

To see the trend of shedding frequency varying with the velocity of drip, numerical simulations are conducted in the Fire Dynamic Simulator (FDS 6.5)^33^ for reference. The model is established in 2-D, and the sizes of drip are 2 mm and 2.6 mm, the same as the experiment. To simulate the dripping process, the position of a circular drip is fixed, while the constant upward flow is provided from the bottom boundary, as illustrated in Fig. 10(b). The upward flow velocity is varied in different cases to simulate different dripping velocities at different heights. The temperature of the drip surface is fixed to its pyrolysis point of 400 °C, and the drip releases the hot inert gas of the same temperature at the flux of 20 g/m^2^-s. To focus on the flow pattern, the chemistry in both gas-phase flame and solid-phase pyrolysis are not included in the model, and Fig. 10(b) uses the temperature contour to illustrate the shedding vortex.

The simulation shows that initially there is a development and transition period before vortex shedding occurs and becomes steady, and the duration of this development period decreases as the flow velocity is increased. As expected, both the modeled vortex shedding frequency (*f*) and the distance between two successive vortices (*l*_*v*_) increase almost linearly with the flow velocity and *Re*^34,35^. Such linearity is characterized by a constant Strouhal number (*St*) under variable dripping velocity (*V*)

14 $$St=\frac{fD}{V}$$

Numerical results show that *St* = 0.40 for *D* = 2 mm and *St* = 0.43 for *D* = 2.6 mm, respectively. In general, there is a reasonable agreement between the frequency of flame shedding in experiment and the frequency of vortex shedding in the model, which supports the proposed hypothesis.

Moreover, this proposed hypothesis is also supported by the unexpected sharp sound during the dripping process (Video 10 in the supplemental material). This special sharp sound only exists when the flame is attached to the drip. After the drip falls for 50 cm, the level of sound increases significantly, as the vortex street starts to develop at a relatively large velocity (or Re number). At the same time, the “*blue chain flame*” can be observed. To collect the sound and study its characteristics, the video camera is placed at 0.5 m and 1.0 m below the source of drip to record the sound and video at the same time. According to the profile of dripping velocity in Fig. 8, for a 4.1-mg drip, the dripping velocity is about 2 m/s at 0.5 m and 3 m/s at 1.0 m, respectively.

The audio process software (Audacity) is used to remove the background noise and apply the fast Fourier transform (FFT) analysis to the sound of more than 20 drips. Figure 10(b) shows the sound frequency spectrum at two sampling locations. For each location, there are two peaks in the spectrum. That is, 320 Hz and 680 Hz at 0.5 m, and 380 Hz and 850 Hz at 1.0 m, respectively. One peak should be the dominant frequency of flame, and the other should be the frequency of vortex street^36^. For a premixed free jet ethylene-air flame in anechoic surroundings, the level of sound is found to peak around 600~900 Hz^37^. Therefore, the second peak in the higher frequency is more likely to be the default sound of premixed flame (or a single ignition). For the first peak, the frequency is not only close to that of flame shedding in Fig. 10(a), but also increases from 320 Hz to 380 Hz, as the height and the velocity of drip are increased. Therefore, the first peak in the lower frequency is most likely to be caused by the flame shedding, i.e., the number of explosion (or ignition) per second.

One question remains, is the premixed vortex ignited by the blue flame in the recirculation zone below or by the previous ignited vortex above? If the previous vortex ignites the new vortex, the velocity of flame propagation should be larger than the velocity of drip. The laminar burning velocity of stoichiometrically mixed ethylene flame in the room temperature is measured to be *S*_*L*_ = 0.67 m/s. If the mixture behind the drip is heated by both the hot drip and the flame, the actual laminar burning velocity could be faster. Assuming second-order reaction, the laminar burning velocity in the preheated mixture^27^ is

15 $$\begin{array}{rcl}{S}_{L}^{^{\prime} } & = & {(\frac{\overline{T}^{\prime} }{\overline{T}})}^{3/8}(\frac{{T}_{u}^{^{\prime} }}{{T}_{u}})(\frac{{T}_{b}}{{T}_{b}^{^{\prime} }})\exp [-\frac{E}{2R}(\frac{1}{{T}_{b}^{^{\prime} }}-\frac{1}{{T}_{b}})]{S}_{L}\\  & = & {(\frac{1500}{1100})}^{3/8}(\frac{800}{300})(\frac{1900}{2200})\exp [-\frac{202\times {10}^{3}}{2\times 8.31}(\frac{1}{2200}-\frac{1}{1900})]\,0.67=4.2\,{\rm{m}}{\rm{/}}{\rm{s}}\end{array}$$

where *E* = 202 kJ/mol and *R* = 8.31 J/mol-K. In the room temperature, *T*_*u*_ = 300 K, *T*_*b*_ = 1900 K, $\overline{T}=1100$ K, while behind the hot drip, ${T}_{u}^{^{\prime} }=800$ K, ${T}_{b}^{^{\prime} }=2200$ K, $\overline{T^{\prime} }=1500$ K may be assumed. The simple calculation indicates that the maximum velocity of flame propagation could be close to the terminal velocity of drip (*V*_*T*_), as shown in Table 1 and Fig. 8. This calculation supports the possibility that the new vortex is ignited by the previously ignited vortex. Nevertheless, it also suggests that extinction will occur if the velocity of drip is much larger than 4 m/s. Because the terminal velocity increases with the size of drip, it also suggests that there may be an upper limit for the size of drip with flame attachment.

### Critical drip size for flame attachment (D_crt_)

Based on the measurement in Fig. 5(c), the critical drip size for flame attachment (*D*_*crt*_) is defined when the probability of flame attachment or igniting a thin tissue paper is larger than 50%, that is, *D*_*crt*_ = 2.3 mm and *M*_*crt*_ = 4.4 mg for a PE drip. Smaller than this critical size, the flame has a larger probability of extinction. The mechanism behind this critical size will be discussed.

One necessary condition for flame attachment is that the flame heating should overcome the environmental cooling and allow the drip to maintain above its pyrolysis point. To simplify the heat transfer process, we assume the top half sphere is heated by the flame, and the bottom half sphere is cooled by air. Thus, the overall flame heating should be larger than the environmental cooling as

16 $${h}_{f}({T}_{f}-{T}_{py})\ge {h}_{c}({T}_{py}-{T}_{a})$$

The convective cooling coefficient (*h*_*c*_) not only increases significantly with the increasing velocity of drip but also with the decreasing drip size (i.e., the curvature effect^38^). Therefore, if the size of the drip is too small or the velocity of the drip is too large, extinction could occur due to the cooling effect. However, this quenching mechanism may not be dominant for the extinction observed in Fig. 7 or responsible for the distribution of extinction height in Fig. 5(a,b). Because the effect of cooling increases linearly with the dripping height, if cooling is the dominant mechanism of extinction, the height of extinction should distribute uniformly throughout the total dripping height of 2.6 m, rather than concentrated within the first 0.7 m as shown in Fig. 5(a,b). Nevertheless, this extinction mechanism due to cooling could become important for a dripping height larger than 2.6 m, which can be further examined in future experiments.

Another necessary condition for flame attachment is that a small diffusion flame should be sustained in the recirculation zone behind the drip. This diffussion flame is very similar to the lifted jet flame^39^, that is, due to the accelecration process of drip, the pyrolysis gas seems to be injected out from the top of drip. Also, such diffussion flame should be able to continously ignite the premixed vortices. If the size of the drip is very small, the recirculation zone behind the drip becomes too small to hold a diffusion flame. Moreover, it is also more difficult for a smaller drip to generate the well-premixed vortex shedding behind the drip under the same velocity of drip, because of the smaller *Re* number (see Eq. (3)). In other words, for a smaller drip, either there is no vortex to ignite, or the vortex is not mixed well enough to reach the flammability limit. Therefore, the flame is not able to stay in the recirculation zone and follow the small drip, but it floats up until burns out, as shown in Fig. 7.

On the other hand, as the size of the drip becomes smaller, the distance between two successive vortices (*l*_*v*_) also becomes smaller^35^. If the previous vortex ignites the new vortex, the flame shedding will be easier to sustain in a smaller drip, which is opposite to the experimental observation. Therefore, the analysis of critical drip size for flame attachment suggests that the premixed vortex is ignited by the blue diffusion flame in the recirculation zone, rather than by the previous ignited vortex.

To better understand the problem, different thermoplastics which have a dripping tendency can be tested to determine the minimum size for dripping (*D*_*min*_) and the critical drip mass (*M*_*crt*_) and size (*D*_*crt*_) for the flame attachment. Materials other than the thin paper (e.g. PMMA and wood) can be examined to further quantify the ignitability of a single drip and multiple drips. Also, new techniques are desired to better control the size and uniformity of drip and produce a large drip (>3 mm) which has a larger terminal velocity.

Future experiments can be conducted in a warehouse with a larger floor height to see if the flame can attach to the drip beyond 2.6 m. It is important to determine if the flame attachment is guaranteed as long as the drip is heavier than 4.4 mg and larger than 2.3 mm, and if there is an upper critical drip size or drip velocity, above which extinction could occur. Furthermore, it is necessary to develop sophisticated numerical models with detailed chemistry in both gas and solid phases to confirm the hypothesis of flame shedding, predict the critical drip size for flame attachment and temperature of drip, and reveal the ignition mechanism of the premixed vortex as well as the key flame chemistry.

## Concluding Remarks

In this work, the drips of molten PE (*D* = 1.9~2.6 mm and *M*_*dr*_ = 2.5~5.0 mg) with flame are produced, and the ignitability, velocity, and flame behaviors of a single drip are investigated. Results suggest the existence of a minimum diameter for dripping (*D*_*min*_), smaller than which the drip will float up rather than fall because of the uprising buoyancy flow induced by the flame of drip. Theoretical calculation shows *D*_*min*_ = 0.63 mm, agreeing well with the experimental measurement.

The necessary condition to ignite the thin tissue paper by a single drip is that the flame must remain attached to the drip at the moment in contact with the paper. The flame is found to either extinguish within the initial 0.7 m or remain attached to the drip, which can ignite the tissue paper, until quenching on the ground 2.6 m below. The probability of flame attachment to drip increases with the size of drip, and the critical mass and diameter of PE drip with flame attachment is found to be *M*_*crt*_ = 4.4 mg and *D*_*crt*_ = 2.3 mm, respectively. Larger than this critical size, the drip can fall with the flame for more than one floor, indicating a significantly greater fire hazard.

The elusive “*blue chain flame*” during the dripping process is observed by human eyes due to the persistence of vision, while the actual “*flame shedding*” process is revealed by the high-speed camera. The flame shedding is most likely to be the continuous ignition of von Karman vortex street generated behind the fast-falling drip. The measured frequency of flame shedding agrees with both the frequency of modeled vortex shedding and the frequency of the unexpected sound. A better understanding of the dripping flame phenomena will helps better evaluate the risk and hazards of wire fire and façade fires. Many aspects of the dripping phenomena are still unclear, such as the ignition mechanism of flame shedding, the maximum velocity and height of dripping for the flame attachment, and the existence of upper drip-size limit for the flame attachment. More experimental and numerical works are desired in future research.

## Electronic supplementary material

Supplemental information

Video 1

Video 2

Video 3

Video 4

Video 5

Video 6

Video 7

Video 8

Video 9

Video 10
