# Supplementary material for: Critical Drip Size and Blue Flame Shedding of Dripping Ignition in Fire
Source: Sci Rep. 2018 Nov 8;8:16528. doi: 10.1038/s41598-018-34620-3 (PMC6224417; doi:10.1038/s41598-018-34620-3)
Supplement: Supplementary file 1 — Supplemental information [file 41598_2018_34620_MOESM1_ESM.docx]

**Critical Drip Size and Blue Flame Shedding of Dripping Ignition in Fire**

**Xinyan Huang**

*Research Center for Fire Engineering, The Hong Kong Polytechnic University, Kowloon, Hong Kong*

xy.huang@polyu.edu.hk

**Supplementary information for videos**

Video 1: Different drips generated from a burning electrical wire.

Video 2: A tissue paper ignited by with a 5-mg drip with flame.

Video 3: A tissue paper not successfully ignited by drips without flame.

Video 4: Dripping process of 5-mg drips with flame attachment (shoot at 60 fps).

Video 5: Dripping process of 5-mg drips with flame attachment (shoot at 120 fps).

Video 6: Dripping process of 5-mg drips with flame attachment (shoot at 960 fps).

Video 7: Dripping process of 2.5 mg drips.

Video 8: The flame extinction process of 2.5-mg drips (shoot at 960 fps).

Video 9: The blue flame on the top of 1.27 cm-thick PMMA rod under an upward airflow of 2.5 m/s.

Video10: Audio samples for the dripping process of 4-mg drips, recorded at different locations.
